# SNP markers reveal relationships between fruit paternity, fruit quality and distance from a cross-pollen source in avocado orchards

**DOI:** 10.1038/s41598-021-99394-7

**Published:** 2021-10-08

**Authors:** Wiebke Kämper, Steven M. Ogbourne, David Hawkes, Stephen J. Trueman

**Affiliations:** 1 Environmental Futures Research Institute, School of Environment and Science, Griffith University, Nathan, QLD Australia; 2 Department of Animal Ecology, Evolution and Biodiversity, Ruhr-University Bochum, Bochum, Germany; 3 GeneCology Research Centre, University of the Sunshine Coast, Maroochydore DC, QLD Australia; 4 Australian Genome Research Facility, Gehrmann Laboratories, University of Queensland, Brisbane, QLD Australia; 5 School of Science, Technology and Engineering, University of the Sunshine Coast, Maroochydore DC, QLD Australia

**Keywords:** Ecology, Molecular biology, Plant sciences, Environmental sciences

## Abstract

Cross-pollination can improve fruit yield, fruit size and nutritional quality of many food crops. However, we rarely understand what proportions of the crop result from self- or cross-pollination, how cross-pollination affects crop quality, and how far pollen is transported by pollinators. Management strategies to improve pollination services are consequently not optimal for many crops. We utilised a series of SNP markers, unique for each cultivar of avocado, to quantify proportions of self- and cross-paternity in fruit of Hass avocado at increasing distances from cross-pollen sources. We assessed whether distance from a cross-pollen source determined the proportions of self-pollinated and cross-pollinated fruit, and evaluated how self- and cross-paternity affected fruit size and nutritional quality. Avocado fruit production resulted from both self- and cross-pollination in cultivar Hass in Queensland, Australia. Cross-pollination levels decreased with increasing distance from a cross-pollen source, from 63% in the row adjacent to another cultivar to 25% in the middle of a single-cultivar block, suggesting that pollen transport was limited across orchard rows. Limited pollen transport did not affect fruit size or quality in Hass avocados as xenia effects of a Shepard polliniser on size and nutritional quality were minor.

## Introduction

Pollination is essential for plant reproduction in both natural and agricultural ecosystems^1–3^. However, plant reproduction is increasingly limited by the quality or quantity of pollen deposited on the stigmas of flowers^4,5^. Roughly 50% of plant species rely on or benefit from cross-pollination for successful reproduction^6,7^. Cross-pollination occurs when pollen from one genotype is transferred to the stigma of another genotype, whereas self-pollination occurs when pollen is transferred within the same genotype^8^. Self-incompatible plants require cross-pollination to reproduce sexually, and some self-compatible plants have increased fruit set when cross-pollinated^9–11^. The type of pollen deposited on the stigma can, thus, be important for successful reproduction^12–14^.

Tree crop orchards are often established with only a few clonally-propagated cultivars. Each cultivar has one genotype, making trees of a single cultivar a clone^8,15^. Cross-pollination occurs when a stigma of one cultivar receives pollen from flowers of another cultivar whereas self-pollination occurs when pollen of the same cultivar is transferred^8^. Cross-pollination increases fruit size, quality and shelf life in many crops, including some tree nuts and berries^14,16–18^. For most crops, we do not understand to what extent self- *v*. cross-pollination contribute to the crop at harvest despite plant reproduction being increasingly pollen limited^4,5^.

Avocado (*Persea americana*, Lauraceae) is a subtropical evergreen tree native to Mexico and Central America. Worldwide, the avocado industry relies on approximately 12 cultivars, but with approximately 90% of production relying on a single cultivar, Hass^19^. Traditionally, avocado cultivars such as the Mexican race including Fuerte had green skins^19^. Nowadays, Hass is often preferred by producers because of its storage and shipping robustness, and by producers and customers because of its change in peel colour from green to black, which covers minor skin imperfections but also provides an index for ripeness^19^. Breeding programs are continuously developing new cultivars, which are often crosses between Hass and another cultivar^19^. Demand for avocado is increasing, partly due to health benefits associated with avocado consumption^20,21^. Avocado fruit are rich in monounsaturated fatty acids such as oleic acid and palmitoleic acid (~ 70%), dietary fibre, vitamins K and E, and the mineral nutrients, potassium and magnesium^20–22^. Consumption of avocado fruit has been linked to improved blood lipid profiles, with lower LDL-cholesterol, lower triglycerides and higher HDL-cholesterol, that are linked to reduced cardiovascular risk^21,23–25^.

Distances between trees of different cultivars in horticultural orchards, including avocado, are often large because single cultivars are planted in wide blocks. This type of planting design can simplify farm management practices^26^. However, planting trees in wide single-cultivar blocks poses a challenge for ensuring cross-pollination. Pollinators must travel long distances across the orchard to transfer pollen between cultivars if distances between the cultivars are large. Cross-pollination is facilitated in crops such as avocado by protogyny, where each flower is functionally female initially and, later, functionally male^27,28^. The flower has six tepals, six stamens, six staminodes and a pistil^28^, but the stamens usually do not open until the day after the receptive stigma has been presented to pollinators. Typically, the flowers either open as female on the morning of the first day, close in the late morning, and then open as male on the afternoon of the following day (type A), or open as female on the afternoon of the first day, close in the late afternoon, and then open as male the following morning (type B). Avocado orchards are established with at least one type A cultivar such as Hass and one type B cultivar such as Shepard to ensure that pollen is available during the female stage of flowers^29^. Trees have profuse flowering and cultivars such as Hass produce many small, seedless fruit that usually abscise very soon after ovule fertilisation^30^. Flies and bees, not wind, are the main pollen vectors in avocado orchards, and most growers introduce managed honey bee hives during flowering to promote pollination^31,32^.

Self-pollination and cross-pollination both occur in avocado^33–35^ and variable outcrossing rates between 31 and 83% have been reported for Hass^33,34,36^. Few studies have assessed the effect of distance from a cross-pollen source on the prevalence of self- *v.* cross-paternity, and very few studies have compared the quality of fruit that resulted from self- *v.* cross-pollination. Consumer desirability might differ between self- and cross-pollinated fruit if the fruit differ in size and nutritional quality. Self-pollinated Fuerte avocado fruit have lower fruit, seed and pericarp mass than cross-pollinated fruit, but similar fruit:seed ratio to cross-pollinated fruit^37^. To our knowledge, no study has investigated the comparative nutritional quality of self- *v.* cross-pollinated avocado fruit.

We aimed to assess the contributions of self- and cross-pollination to fruit production in avocado orchards. We expected pollen to be transported over short distances. We hypothesised that there would be a greater proportion of self-pollinated fruits with increasing distance from a cross-pollen source and that self-pollination would decrease fruit size and quality. We aimed to determine how far cross-pollen was effectively transferred across orchard rows. We aimed to quantify whether the proportions of self- and cross-pollinated fruit, fruit size (including fruit mass, flesh mass and seed mass) and seed proportion, varied at different distances from a cross-pollen source. We also aimed to identify how self- and cross-pollination affected fruit size, seed proportion, mineral nutrient concentrations and fatty acid composition of avocado fruit flesh.

## Results

### Paternity and fruit size at different distances from a cross-pollen source

**Figure 1:**
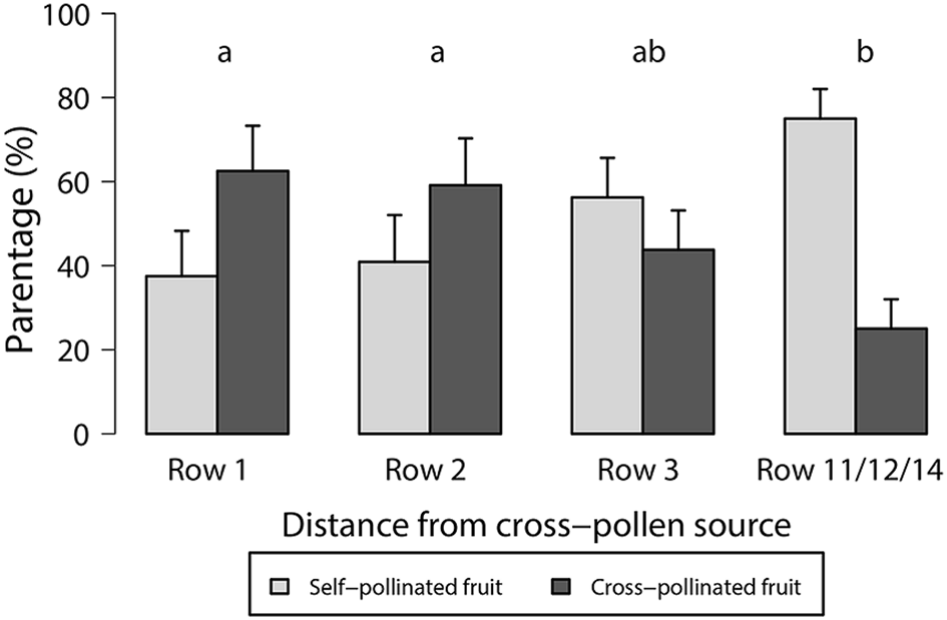
Percentage of cross-pollinated and self-pollinated Hass avocado fruits at different numbers of rows from a cross-pollen source. Fruits were sampled along transects starting at trees adjacent to another cultivar (Row 1) and ending in the middle row of the Hass block (Row 11, 12 or 14). Means (+ SE) for cross-parentage and self-parentage with different letters are significantly different (two-way ANOVA and Tukey’s HSD; *P* < 0.05; n = 8).

A total of 52.4% of Hass fruit (*N* = 190) resulted from self-pollination and 47.6% resulted from cross-pollination in Queensland, Australia. Almost all the cross-pollinated fruit (95%; 86 of 91 fruit) were pollinated by cultivar Shepard. The remaining five cross-pollinated fruit were pollinated by Lamb Hass (1 fruit) or Sharwil (1 fruit) or the cross-pollen parent could not be assigned definitively (3 fruit). The percentage of self-pollinated fruit increased with increasing distance (i.e. the number of rows) from a cross-pollen source, from 37 to 75%, whereas the percentage of cross-pollinated fruit decreased from 63 to 25% (Fig. 1; *F* = 4.00, *P* = 0.02). The percentage of cross-pollinated fruit declined significantly by 11 rows from a cross-pollen source (Fig. 1). Distance from a cross-pollen source did not affect fruit mass (*F* = 0.39, *P* = 0.76), flesh mass (*F* = 0.60, *P* = 0.62), seed mass (*F* = 0.72, *P* = 0.55) or seed proportion (*F* = 2.48, *P* = 0.09) (Table 1).

**Table 1:** Fruit size of avocado cultivar Hass fruit collected at different distances from a cross-pollen source.

Fruit size	Row 1	Row 2	Row 3	Row 11/12/14
Total fresh mass (g)	193.2 ± 12.0	190.8 ± 13.6	207.6 ± 2.8	201.9 ± 15.6
Flesh mass (g)	153.7 ± 8.8	159.8 ± 13.3	168.1 ± 2.4	166.7 ± 14.4
Seed mass (g)	39.5 ± 3.3	34.9 ± 3.5	39.5 ± 2.4	36.6 ± 2.3
Seed proportion	20.4 ± 0.7	18.7 ± 0.7	18.9 ± 1.1	18.4 ± 0.7

Means ± SE within each parameter do not differ significantly (mixed model; *P* > 0.05; n = 32 trees).

### Effect of pollen parentage on fruit size, mineral nutrient concentrations and fatty acid composition

Hass avocado fruit that were self-pollinated by Hass or cross-pollinated by Shepard did not differ significantly in fruit mass, flesh mass, seed mass or seed proportion (Table 2). Self-pollinated fruit had 9.1% lower calcium and 10.6% higher phosphorus concentrations than cross-pollinated fruit (Table 3). Self- and cross-pollinated fruit did not differ in the concentrations of other elements (Table 3). Self-pollinated fruit had a 4.4% higher ratio of unsaturated to saturated fatty acids (UFA:SFA) than cross-pollinated fruit (Table 4). Self- and cross-pollinated fruit did not differ significantly in the relative contributions of palmitic, palmitoleic, stearic, oleic, elaidic or linoleic acid to the total fatty acid composition (Table 4).

**Table 2:** Fruit size of avocado cultivar Hass fruit pollinated by self-pollen (Hass) or cross-pollen (Shepard).

Fruit size	Pollen parent	
	Hass (self-pollinated)	Shepard (cross-pollinated)
	N = 99	N = 86
Total fresh mass (g)	195.5 ± 6.0	197.8 ± 8.3
Flesh mass (g)	155.8 ± 4.6	161.0 ± 6.7
Seed mass (g)	37.2 ± 1.5	37.0 ± 1.9
Seed proportion	18.5 ± 0.5	19.2 ± 0.5

Means ± SE do not differ significantly (mixed model; *P* > 0.05; n = 32 trees).

**Table 3:** Nutrient concentrations in the flesh of avocado cultivar Hass fruit pollinated by self-pollen (Hass) or cross-pollen (Shepard).

Nutrient	Pollen parent	
	Hass (self-pollinated)	Shepard (cross-pollinated)
	N = 95	N = 83
C (%)	16.0 ± 0.3	15.7 ± 0.3
N (%)	0.20 ± 0.01	0.17 ± 0.01
Al (mg/kg)	4.1 ± 0.9	3.9 ± 1.0
B (mg/kg)	32.5 ± 2.2	34.1 ± 3.2
Ca (mg/kg)	131.8 ± 9.5b	144.9 ± 11.4a
Cu (mg/kg)	3.5 ± 0.2	3.3 ± 0.2
Fe (mg/kg)	10.3 ± 0.7	9.8 ± 1.0
K (mg/kg)	4929.1 ± 185.6	4719.2 ± 227.8
Mg (mg/kg)	277.6 ± 9.3	281.6 ± 12.7
Mn (mg/kg)	2.4 ± 0.1	2.5 ± 0.2
Na (mg/kg)	152.0 ± 9.5	151.2 ± 7.8
P (mg/kg)	515.6 ± 18.6a	461.2 ± 20.1b
S (mg/kg)	320.1 ± 16.3	297.2 ± 19.4
Zn (mg/kg)	10.0 ± 0.6	9.2 ± 0.8

Means ± SE with different letters are significantly different (mixed model; *P* < 0.05; n = 32 trees).

**Table 4:** Relative abundances of fatty acids (mean ± SE) in the flesh of avocado cultivar Hass fruit pollinated by self-pollen (Hass) or cross-pollen (Shepard).

Fatty acid	Pollen parent	
	Hass (self-pollinated)	Shepard (cross-pollinated)
	N = 96	N = 83
Palmitic—C16:0 (%)	31.8 ± 0.9	32.1 ± 0.9
Palmitoleic—C16:1 cis (%)	11.6 ± 0.7	11.9 ± 0.5
Stearic—C18:0 (%)	0.4 ± 0.0	0.4 ± 0.0
Oleic—C18:1 *cis* (%)	40.0 ± 1.3	39.1 ± 1.0
Elaidic—C18:1 *trans* (%)	7.0 ± 0.3	7.1 ± 0.3
Linoleic—C18:2 (%)	9.2 ± 0.5	9.4 ± 0.5
Saturated fatty acids (SFA)	31.9 ± 0.9	32.7 ± 1.0
Unsaturated fatty acids (UFA)	68.1 ± 0.9	67.3 ± 1.0
UFA:SFA	2.17 ± 0.09a	2.08 ± 0.09b

Means ± SE with different letters are significantly different (mixed model; *P* ≤ 0.05; n = 32 trees).

## Discussion

Self- and cross-paternity were both common among Hass avocado fruit in our study, but the percentage of cross-pollinated fruit decreased with increasing distance from a cross-pollen source. Self- and cross-pollinated fruit did not differ significantly in fruit size and they differed little in their nutritional composition. However, self-pollinated fruit had lower calcium concentrations, higher phosphorus concentrations and a slightly elevated UFA:SFA ratio. These results demonstrate that pollen flow is limited across avocado orchards, but that xenia effects of a Shepard polliniser on the size and nutritional quality of Hass fruit quality are minor.

Hass avocado fruit resulted from either self-pollination or cross-pollination in Queensland, Australia, demonstrating some degree of self-compatibility in avocado flowers. Between 25 and 63% of fruit were cross-pollinated, with the frequency of outcrossing being dependent on the proximity to flowers of another cultivar. The 25% of fruit that were cross-pollinated in the middle of a Hass block had received pollen that was transported at least 130–147 m, even though self-pollen was potentially available from all trees planted at a closer distance. The orchards in our study were established with Hass, Lamb Hass and Wurtz as type A cultivars and Shepard as the type B cultivar, ensuring that Shepard pollen was available during the female stage of the Hass, Lamb Hass and Wurtz trees. Protogyny can explain why 25% of fruit were cross-pollinated in the middle of the single-cultivar Hass block because only cross pollen was widely released during the female stage of Hass flowers. The temporal separation between male and female flowers in the flowering types is not complete and can be affected by temperature^38^. Other mechanisms such as selective fruitlet abscission might also have occurred^34,39^. Self-pollinated avocado fruitlets can be more prone to selective abortion, leading to an over-representation of cross-pollinated fruit at harvest^39^. However, younger fruitlets resulting from pollination late in flowering are also more prone to selective abortion^34^.

A decrease in the percentage of cross-pollinated Hass fruit with increasing distance from a cross-pollen source has been reported previously^34,40^. Our results showed a significant decrease in the percentage of cross-pollinated fruit when comparing the middle of a single-cultivar block to the row adjacent to a cross-pollen source, but not when comparing 2 or 3 rows from the cross-pollen source to the row adjacent to the cross-pollen source. Other studies have reported a decrease in the percentage of cross-pollinated fruit at smaller distances from a cross-pollen source than we found; e.g. from 0 to 28 m or from 0 to 92 m compared with from 0 to 130 m in our study^34,40^. The high percentages of cross-pollinated fruit at 2 or 3 rows from a cross-pollen source in our study may suggest that Shepard pollen is moved further across orchard rows or it may suggest differences in pollinator movements compared with other studies^41–43^.

Cross-pollination increases fruit quality, such as size and nutritional composition, of many crops^5,16–18^. However, we found that self- and cross-pollinated avocado fruit did not differ in fruit mass, flesh mass, seed mass or seed proportion. This contradicts previous studies which found fruit mass and seed mass of cross-pollinated avocado fruit were higher than those of self-pollinated fruit^39,44^. Fuerte avocado fruit cross-pollinated by Tops-Tops, Teague or Ettinger have higher fruit mass and seed mass than self-pollinated fruit^39^. Hass fruit cross-pollinated by Ettinger have higher seed mass than self-pollinated fruit^44^. The previous study on Hass fruit and our study investigated different cross-pollen parents. It is possible that the observed difference is caused by different pollen parents, where pollination by Ettinger results in larger Hass seed mass whereas pollination by Shepard investigated in our study did not.

For most crops, we do not know how self- v. cross-pollination affect crop quality parameters such as nutritional quality, and whether an altered nutritional quality of the fruit can provide health benefits for consumers or change postharvest properties. Fatty acid composition and nutrient concentrations differed little between self- and cross-pollinated Hass fruit. However, self-pollinated fruit had a higher UFA:SFA ratio than cross-pollinated fruit. To our knowledge, no studies have investigated the nutritional quality of self- and cross-pollinated avocado fruit. The slightly higher UFA:SFA ratio of self-pollinated fruit could be beneficial for human health because a diet rich in unsaturated fatty acids decreases LDL-cholesterol levels and other cardiovascular risk factors^21,25,45^. Cross-pollination in almonds increases the oleic to linoleic acid ratio, which has been linked to their cardio-protective effects^17,46^. However, the UFA:SFA ratio in avocado is highly variable and the ratio in our study was relatively low at 2.17 and 2.08 in self- and cross-pollinated fruit, respectively^21,47^. The consumption of fruit from cooler growing regions that have higher UFA:SFA ratios will have a much greater effect on health than the small observed difference between self- and cross-pollinated fruit in our study^47^. Self-pollinated fruit had 9% lower calcium and 11% higher phosphorus concentrations than cross-pollinated fruit. Phosphorus, unlike other micronutrients such as calcium, iron, iodine, magnesium and zinc, whose dietary intakes are often inadequate, is almost never in short supply in the human diet^48^. The calcium nutrient levels of cross-pollinated fruit might be more beneficial for human health. Calcium-deficiency has been linked to many physiological disorders^49^. Calcium levels in avocado fruit are also critical for storage and transport because avocado fruit high in calcium ripen more slowly and have prolonged shelf life^50^, likely because calcium is important for cell wall rigidity^51^.

Self- and cross-pollination both contributed to the harvested Hass avocado crop in Queensland, Australia. The percentage of cross-pollinated fruit decreased with increasing distance from a cross-pollen source, indicating limited cross-pollen movement to the middle of single-cultivar blocks. Xenia effects of a Shepard polliniser were minimal, because fruit size, the levels of most mineral nutrients, and the contributions of most fatty acids to total fatty acid composition did not differ significantly between self- and cross-pollinated fruit. However, further research should be undertaken to determine whether cross-pollination affects initial fruit set, fruitlet retention and, thus, overall tree yield.

## Material and methods

### Study sites and design

Hass avocado fruit were harvested from two commercial orchards near Childers, Queensland, Australia (25°08′ 17′′ S 152° 22′ 40′′ E and 25° 13′ 32′′ S 152° 17′ 53′′ E). The collection of plant material complied with institutional, national, and international guidelines and legislation. The soil at both orchards was red clay-loam. The average maximum daily temperature during the study (April and May 2018) was 27.2 °C, the average minimum daily temperature was 16.3 °C and the total rainfall was 36 mm (Bureau of Meteorology, Bundaberg, 2020). Orchard 1 contained blocks of Hass and Shepard that were 26 rows wide, with trees being 13 years old. Tree spacing was 10–11 m between rows and 5 m within a row. The distance to the next block containing trees of the other cultivar was 15–20 m because blocks of different cultivars were separated by roads. Orchard 2 contained blocks of Hass, Shepard, Lamb Hass and Wurtz that were 6–22 rows wide, with trees being 18–21 years old. Tree spacing was 10 m between rows and 5 m within a row. The distance to the next block containing trees of a different cultivar was 10 m.

A total of 320 mature Hass fruit was collected from 32 trees from two transects in orchard 1 and six transects in orchard 2. Each transect consisted of individual trees at four sampling points: (a) 1, (b) 2, (c) 3 and (d) 11–14 rows from the cross-pollen source. The last sampling point was chosen to represent the middle of the block, and thus depended on the width of the single-cultivar block. Ten fruit were collected in a stratified design from each tree, with each tree divided into five sectors on the side of the tree that faced the neighbouring cultivar^43^. Two fruit were sampled per sector, one from the inside and one from the outside of the canopy, on either 18 April or 9/10 May 2018, depending on the orchard. Six fruit per tree were selected randomly for further analyses, resulting in 192 samples (48 samples per sampling point at 1, 2, 3 and 11–14 rows from the cross-pollen source). Fruit were kept in the shade until moved to a cold room at 4 °C within 20 h of collection^52,53^.

Fruit were stored at 4 °C for 10 or 20 d, before being moved to room temperature (21 °C) to allow onset of ripening. Fruit were ripe after 10.6 ± 1.0 days (mean ± SE) at room temperature. Ripeness was confirmed by measuring skin and flesh firmness with a handheld sclerometer (8 mm head; Lutron Electronic Model: FR-5120, Coopersburg, PA). Fruit were considered ripe when the maximum force required to impress the sclerometer tip 1 mm deep was < 15 N for the skin and < 5 N for the flesh^54,55^. Flesh firmness was measured after removing small patches of skin at two locations along the equator of the fruit, with the two measurements taken at 90° from each other. Fruit and seed fresh mass were recorded. Subsamples of flesh were then taken to measure the: (1) relative contribution of six fatty acids to the total fatty acid composition; (2) ratios of saturated and unsaturated fatty acids; and (3) concentrations of mineral nutrients. A ~ 50 mg subsample of the seed was taken for genotyping.

### Mineral nutrients

We determined the concentrations of 14 nutrients from flesh taken from two locations, near the apex and along the equator of each fruit. We used combustion analysis (TruSpec, LECO Corporation, St. Joseph, MI) for nitrogen (N) and carbon (C) and inductively coupled plasma–atomic emission spectroscopy (Vista Pro, Varian Incorporation, Palo Alto, CA) after nitric and perchloric acid digestion for sulphur (S), phosphorus (P), potassium (K), aluminium (Al), boron (B), calcium (Ca), copper (Cu), iron (Fe), magnesium (Mg), manganese (Mn), sodium (Na) and zinc (Zn)^56–59^.

### Fatty acids

Approximately 40 g of flesh was taken from each fruit and oil was extracted using a modification of the protocol of Bai et al.^60^. The flesh was mashed finely before being stirred for 15 min in the presence of 30 mL of pentane. Fatty acid composition was determined by gas chromatography–mass spectrometry. Peak areas were used to calculate the relative proportions of each fatty acid. Fatty acids that consistently accounted for < 0.3% of the composition were excluded.

### MassARRAY design

DNA extraction followed the glass-fibre plate DNA extraction protocol for plants (http://ccdb.ca/resources/) ^61^. We used disposable 2.3 mm and 0.1 mm zirconia/silica beads prior to shaking on a TissueLyser II (Qiagen, Hilden, Germany). A double-digest RADseq approach (ddRADseq) was used to screen 42 samples from 10 avocado cultivars for private alleles: Carmen Hass (leaf samples of 5 individual trees), Fuerte (4), Hass (5), Lamb Hass (5), Maluma Hass (5), Reed (4), Sharwil (3), Shepard (5), Velvick (1), and Wurtz (5)^62,63^. The highly-similar cultivars Hass and Carmen Hass produced no private alleles, and so the analysis was performed treating Hass and Carmen Hass as a single group.

Is commonly performed using 75 bp reads. We opted for longer reads (150 bp) to support downstream assay development for MassARRAY genotyping assays. Sequences extracted for private alleles from each cultivar were imported into Agena Assay Design Suite 2 (www.agenobio.com). All proximal variants identified by Stacks were annotated onto the sequences, and preference was given to sequences with low degrees of variation. Standard design parameters were used except for the following changes to improve multiplexing: false priming threshold (0.8), primer dimer threshold (0.8), amplicon length variation (0.9), PCR primer T_m_ variation (0.9), maximum pass iteration base (200). The design produced a single multiplex containing primer pairs and extension primers for 28 assays (Supplementary Material S1 and S2).

### MassARRAY genotyping

High-throughput genotyping was performed using the Agena MassARRAY platform (Agena Bioscience, San Diego, CA, USA) to assign paternity of avocado seeds. Briefly, the extracted avocado seed DNA (2 uL; ~ 10 ng/ul) was amplified in 5 uL multiplex PCR reactions containing 1 U of Taq, 2.5 pmol of each PCR primer, and 500 μM of each dNTP (PCR Accessory and Enzyme Kit, Agena). Thermocycling was performed at 94 °C for 4 min followed by 45 cycles of 94 °C for 20 s, 56 °C for 30 s, and 72 °C for 1 min, and a final extension at 72 °C for 3 min. Unincorporated dNTPs were deactivated using 0.5 U of shrimp alkaline phosphatase (37 °C for 4 min, 85 °C for 5 min). Primer extension was initiated by adding 1.3 U of iPLEX GOLD, dideoxy nucleotide terminators and extension primers. The reaction conditions consisted of 95 °C for 30 s, 40 cycles of 95 °C for 5 s plus five inner cycles of 52 °C for 5 s and 80 °C for 5 s, and a final extension at 72 °C for 3 min. A cation exchange resin was added to remove residual salt, and 7 nL of the purified primer extension product was loaded onto the matrix pad of a SpectroCHIP (Agena) using an RS1000 nanodispenser. The extension products were analysed by matrix assisted laser desorption ionization-time of flight mass spectrometry (MALDI-TOF MS - 4300 to 9000 Daltons) using TYPER Analyzer 4.0 software (www.agenabio.com) to identify the alleles and to genotype the samples. A total of 98% of samples could be assigned by mass array.

We cross-validated the mass array results with pre-published microsatellite markers^64,65^. Details on the microsatellite markers and laboratory protocols are presented in Supplementary Material S3. The microsatellite markers could only assign 74% of the samples, 62% of which could be assigned with 95% statistical confidence and another 38% only with 80% statistical confidence. The novel SNP markers could assign parentage to the complete set of samples with 100% statistical confidence. Of the 74% of samples that could be assigned with microsatellites 98% agreed with the mass array assignment.

### Statistical analyses

We calculated the proportions of cross-pollinated and self-pollinated fruit per tree. We used two-way ANOVA to test whether distance from a cross-pollen source (measured as number of rows) and orchard affected the proportion of cross-pollinated and self-pollinated fruit. The interaction between distance and orchard was not significant. We used linear mixed models with tree number, transect and orchard as random effects to test whether distance from a cross-pollen source, as a fixed and categorical variable, affected fruit mass, flesh mass, seed mass and seed proportion. Flesh mass was calculated by subtracting seed mass from the fruit mass, and seed proportion was calculated as the amount of fruit mass that consisted of seed mass. Tukey's all-pair comparisons tests were performed when differences were detected.

We also used linear mixed models with tree number, transect and orchard as random effects to compare size and nutritional quality between self-pollinated fruit and fruit that were cross-pollinated by the predominant polliniser, Shepard. We compared (1) fruit mass, (2) flesh mass, (3) seed mass, (4) seed proportion, (5) concentrations of each of 14 mineral nutrients, (6) relative contributions of six fatty acids to the total fatty acid composition, (7) relative contributions of saturated and unsaturated fatty acids to the total fatty acid composition, and (8) ratio of unsaturated to saturated fatty acids. Data was log-transformed before analysis when necessary to achieve normal data distribution. Tukey's all-pair comparisons tests were performed when differences were detected.

Statistical analyses were performed using R version 3.1.1 for Macintosh OS X^66^. Mixed models were performed with the package ‘lmerTest’ and ‘multcomp’ in R^67^.

## Supplementary Information

Supplementary Information 1.

## Data Availability

Data used in analyses are uploaded as supplementary material.
