# Supplementary material for: SNP markers reveal relationships between fruit paternity, fruit quality and distance from a cross-pollen source in avocado orchards
Source: Sci Rep. 2021 Oct 8;11:20043. doi: 10.1038/s41598-021-99394-7 (PMC8501009; doi:10.1038/s41598-021-99394-7)
Supplement: Supplementary file 1 — Supplementary Information 1. [file 41598_2021_99394_MOESM1_ESM.docx]

Supplementary materials for the article

**SNP markers reveal relationships between fruit paternity, fruit quality and distance from a cross-pollen source in *Persea americana* orchards**

Wiebke Kämper^*^, Steven M. Ogbourne, David Hawkes, Stephen J. Trueman

*Corresponding author: w.kaemper@griffith.edu.au

| **Table S1** 28 MassARRAY genotyping assays. Sequences are stored in BankIt. | | | | | | | | | | | |  | | | |  | | | |
| --- | --- | --- | --- | --- | --- | --- | --- | --- | --- | --- | --- | --- | --- | --- | --- | --- | --- | --- | --- |
| Assay | | | Loci | REF | | ALT | | PCR1 | | | | PCR2 | | | | EXT | | | |
| FUERTE_46273_C | | | 46273 | C | | T | | ACGTTGGATGCACGAAATATACCCCTTGAG | | | | ACGTTGGATGTGAGGAAGAAGAAGGTAGGC | | | | GGCGGAGAATAGATTCA | | | |
| FUERTE_52667_C | | | 52667 | C | | T | | ACGTTGGATGCCCAAAGTGGGTATCCAAAG | | | | ACGTTGGATGAATCGATCTATGGGCCTAAC | | | | CCCCATATGGGCCTAACATGACTCC | | | |
| FUERTE_93565_T | | | 93565 | T | | C | | ACGTTGGATGCCTGTAAATGCCGGCTTTCC | | | | ACGTTGGATGCGGAGCACCAAATAATTCCC | | | | AAAATGTACATGTGGTTCACC | | | |
| HASS_CARMENHASS_73255_GRPb_A | | | 73255 | A | | T | | ACGTTGGATGCAATAGTAACATATTCTACTG | | | | ACGTTGGATGCAGTCTTATATGAGAGGAGG | | | | GGAGAAATTGTTTTGCCA | | | |
| HASS_CARMENHASS_84299_GRPb_T | | | 84299 | T | | T | | ACGTTGGATGACAAGAGCCTGCATCTTGAG | | | | ACGTTGGATGCCTCTCTCATCACCAACAAG | | | | GCGATCAGTTCCTCC | | | |
| LAMBHASS_20958_G | | | 20958 | G | | A | | ACGTTGGATGGTTGGTTCTGAAGAAAGATG | | | | ACGTTGGATGAACCTGCTTCATGTTGTGAG | | | | GGGGCGAAACICCTCCTTGCT | | | |
| LAMBHASS_45259_T | | | 45259 | T | | A | | ACGTTGGATGGTGGAAGGGCACTTCTAAAG | | | | ACGTTGGATGTCIGCTGAGAATGTATCCTG | | | | CAGTTCTTTAGTTACAAACCCC | | | |
| LAMBHASS_93019_G | | | 93019 | G | | A | | ACGTTGGATGTGGTTGGTAATGGTGCATGG | | | | ACGTTGGATGGGACAGTTGATTGCTCTATG | | | | GGTGATGATGATGCTACCC | | | |
| MALUMA_18696_C | | | 18696 | C | | T | | ACGTTGGATGGGATGTAATGATCTTGGCTC | | | | ACGTTGGATGTGGTTTTCITTCTCAAGTGG | | | | TGAGAAAAAGAGGGAAAAGAAA | | | |
| MALUMA_23841_G | | | 23841 | G | | A | | ACGTTGGATGTGGAGAAAACCCATCACCTG | | | | ACGTTGGATGTTGGATCACTCAATAGCGGG | | | | GAACCTGGATGTAGACTAATGTAATG | | | |
| MALUMA_82866_G | | | 82866 | G | | A | | ACGTTGGATGCAATGGGGAAAACACACAGC | | | | ACGTTGGATGAAGGACACAGCAGACAAGAG | | | | ACTCAAAACCCTCGGG | | | |
| MALUMA_93949_T | | | 93949 | T | | C | | ACGTTGGATGGAGAATATGGAGCAGGTCAC | | | | ACGTTGGATGATGCTGCATCATGTGTGCTG | | | | GGCAGTTGATAGGATAGAATAATAGT | | | |
| REED_31271_A | | | 31271 | A | | T | | ACGTTGGATGAGICTCACATGAGCCTATGC | | | | ACGTTGGATGAGGGCTTAGAAACCCCATTG | | | | GGGAGTGGTCCAGGCAATAAA | | | |
| REED_81676_C | | | 81676 | C | | T | | ACGTTGGATGCCTTCTCAAATATTTAGCTCG | | | | ACGTTGGATGGCCACACGAGTACTGTTTTG | | | | CCCCACGTTTTCGGC | | | |
| REED_92177_T | | | 92177 | T | | A | | ACGTTGGATGTCCTGGCCAGGATTTCATTG | | | | ACGTTGGATGTGACATATTCCAACAATCG | | | | TTCCAACAATCGCTGTAG | | | |
| SHARWIL_116939_G | | | 116939 | G | | T | | ACGTTGGATGGGCCACATAGTCCAAGTATC | | | | ACGTTGGATGACGTGGCTCTCCTAAAAGTC | | | | TCCAAAAATTTCCACCC | | | |
| SHARWIL_32517_A | | | 32517 | A | | T | | ACGTTGGATGAGCAACCTGAAAAAAGAGAG | | | | ACGTTGGATGTCAGAGCACAACATCAGAAC | | | | CTGTATAGTTGTAATAAAGAAATGG | | | |
| SHARWIL_43380_G | | | 43380 | G | | A | | ACGTTGGATGGAAGCTTTTCGGGAGGTTGG | | | | ACGTTGGATGTCGATTTGGACATCTGGCTC | | | | CGCCACTTGCTAGACTTGTC | | | |
| SHARWIL_67950_C | | | 67950 | C | | T | | ACGTTGGATGGAATGTTTACGGACAGTCGG | | | | ACGTTGGATGGCATTTGTCAAAGGITCTCC | | | | GGTGTACAAGAAATGTCAG | | | |
| SHEPHARD_111476_C | | | 111476 | C | | G | | ACGTTGGATGGGAGGTGAAAAGGAAAAGCG | | | | ACGTTGGATGGTCAAGGATCCATGGAAAAG | | | | TAAGAACATAAAGAATATACAGTTG | | | |
| SHEPHARD_4390_G | | | 4390 | G | | A | | ACGTTGGATGTTGTGCTATCTATGTGGTC | | | | ACGTTGGATGCACACTCATCTCAACCATCC | | | | ACATGATAAAACACAGTTAGC | | | |
| SHEPHARD_91293_T | | | 91293 | T | | C | | ACGTTGGATGCCTGGGTTGTATTTTATCTG | | | | ACGTTGGATGCCGTGTCTATATTTGGATGC | | | | CGGGACCTGAIAACAGCAACCAAA | | | |
| VELVICK_94477_G | | | 94477 | G | | T | | ACGTTGGATGATCTTCTTTGCCCACCCAAG | | | | ACGTTGGATGGGAAAGGCTTATCCTCTTGG | | | | CCTCTTGGAATACTTTATCAAATATA | | | |
| VELVICK_94905_C | | | 94905 | C | | G | | ACGTTGGATGCTACTTGGACCAACGAATGC | | | | ACGTTGGATGGGAAATAACCATCTGCTGGG | | | | GACGGGCAGTTTGTCTTAGG | | | |
| VELVICK_9837_G | | | 9837 | G | | A | | ACGTTGGATGTGCAGAATCAGCAACATGGG | | | | ACGTTGGATGCAGGAAATGCTTGCATTGAG | | | | CCTGAGATCTCAGTATCCAAAAA | | | |
| WURTZ_21778_C | | | 21778 | C | | T | | ACGTTGGATGATAGCCATAGCCCATGACTG | | | | ACGTTGGATGATGGGAAAGACTATGGCCT | | | | AGTTACCTTGACTGITAC | | | |
| WURTZ_2603_C | | | 2603 | C | | T | | ACGTTGGATGCCGAATAGACACTTCTTGGC | | | | ACGTTGGATGAACTAAATGCTATGTCTAC | | | | CCTGCTATGTCTACGTTGTC | | | |
| WURTZ_61596_A | | | 61596 | A | | C | | ACGTTGGATGCCTTCCCCTCTTCTTTTTTTC | | | | ACGTTGGATGGGCTTTTGTTGCCCTACTTC | | | | ACAGAAGGATCGAGAAAAAG | | | |
| REF Polymorphism in target cultivar, ALT Polymorphism in other cultivars | | | | | | | | | | | | | | | | | | | |
| **Table S1 (continued)** | |  | | |  | |  | |  |  |  | |  |  |  | |  |  |  |
| Assay | Loci | REF | | | ALT | | Fuerte | | Hass | Hass Carmen | Lamb Hass | | Maluma | Reed | Sharwil | | Shepard | Velvick | Wurtz |
| FUERTE_46273_C | 46273 | C | | | T | | CC | | TT | TT | TT | | TT | TT | TT | | TT | TT | TT |
| FUERTE_52667_C | 52667 | C | | | T | | CC | | TT | TT | TT | | TT | TT | TT | | TT | TT | TT |
| FUERTE_93565_T | 93565 | T | | | C | | TT | | CC | CC | CC | | CC | CC | CC | | CC | CC | CC |
| HASS_CARMENHASS_73255_GRPb_A | 73255 | A | | | T | | TT | | AA | AA | TA | | TT | TA | TT | | TT | TT | TT |
| HASS_CARMENHASS_84299_GRPb_T | 84299 | T | | | T | | GG | | TT | TT | GT | | GG | GG | GG | | GG | GT | GT |
| LAMBHASS_20958_G | 20958 | G | | | A | | AA | | AA | AA | GG | | AA | AA | AA | | AA | AA | AA |
| LAMBHASS_45259_T | 45259 | T | | | A | | AA | | AA | AA | TT | | AA | AA | AA | | AA | AA | AA |
| LAMBHASS_93019_G | 93019 | G | | | A | | AA | | AA | AA | GG | | AA | AA | AA | | AA | AA | AA |
| MALUMA_18696_C | 18696 | C | | | T | | TT | | TT | TT | TT | | CC | TT | TT | | TT | TT | TT |
| MALUMA_23841_G | 23841 | G | | | A | | AA | | AA | AA | AA | | GG | AA | AA | | AA | AA | AA |
| MALUMA_82866_G | 82866 | G | | | A | | AA | | AA | AA | AA | | GG | AA | AA | | AA | AA | AA |
| MALUMA_93949_T | 93949 | T | | | C | | CC | | CC | CC | CC | | TT | CC | CC | | CC | CC | CC |
| REED_31271_A | 31271 | A | | | T | | TT | | TT | TT | TT | | TT | AA | TT | | TT | TT | TT |
| REED_81676_C | 81676 | C | | | T | | TT | | TT | TT | TT | | TT | CC | TT | | TT | TT | TT |
| REED_92177_T | 92177 | T | | | A | | AA | | AA | AA | AA | | AA | TT | AA | | AA | AA | AA |
| SHARWIL_116939_G | 116939 | G | | | T | | TT | | TT | TT | TT | | TT | TT | GG | | TT | TT | TT |
| SHARWIL_32517_A | 32517 | A | | | T | | TT | | TT | TT | TT | | TT | TT | AA | | TT | TT | TT |
| SHARWIL_43380_G | 43380 | G | | | A | | AA | | AA | AA | AA | | AA | AA | GG | | AA | AA | AA |
| SHARWIL_67950_C | 67950 | C | | | T | | TT | | TT | TT | TT | | TT | TT | CC | | TT | TT | TT |
| SHEPHARD_111476_C | 111476 | C | | | G | | GG | | GG | GG | GG | | GG | GG | GG | | CC | GG | GG |
| SHEPHARD_4390_G | 4390 | G | | | A | | AA | | AA | AA | AA | | AA | AA | AA | | GG | AA | AA |
| SHEPHARD_91293_T | 91293 | T | | | C | | CC | | CC | CC | CC | | CC | CC | CC | | TT | CC | CC |
| VELVICK_94477_G | 94477 | G | | | T | | TT | | TT | TT | TT | | TT | TT | TT | | TT | GG | TT |
| VELVICK_94905_C | 94905 | C | | | G | | GG | | GG | GG | GG | | GG | GG | GG | | GG | CC | GG |
| VELVICK_9837_G | 9837 | G | | | A | | AA | | AA | AA | AA | | AA | AA | AA | | AA | GG | AA |
| WURTZ_21778_C | 21778 | C | | | T | | TT | | TT | TT | TT | | TT | TT | TT | | TT | TT | CC |
| WURTZ_2603_C | 2603 | C | | | T | | TT | | TT | TT | TT | | TT | TT | TT | | TT | TT | CC |
| WURTZ_61596_A | 61596 | A | | | C | | CC | | CC | CC | CC | | CC | CC | CC | | CC | CC | AA |

| **Table S1 (continued)** |  |
| --- | --- |
| Assay | Sequence |
| FUERTE_46273_C | TGCAGAGAACAACAATGAATGATGAGGAAGAAGAAGGTAGGCGGAGAATAGATTCA[T/C]CGAGAGACTTTTTATTGTACTTTTTTTTCTTTCTCAAGGGGTATATTTCGTGTAAAACGATCAATCTGTGAAGTGGCCCATTTA |
| FUERTE_52667_C | TGCAGCCCAAAGTGGGTATCCAAAGTAAATAGGAAAATGGCTAGAATTGGTTAGATAATGTTTCAGCCTAGCAAG[T/C]GGAGTCATGTTAGGCCCATAGATCGATTCGACAGTTCTTCACATCATGCCACGACAATGAGGTT |
| FUERTE_93565_T | TGCAGCCTGTAAATGCCGGCTTTCCGATGATTT[C/T]GGTGAACCACATGTACAGGTATAGAGTGGTGGGAATTATTTGGTGCTCCGCTGGAGTTTGTGAATTATGGATGTAAATGATGGAAAGTTGGGTAAATGTTTCACAT |
| HASS_CARMENHASS_73255_GRPb_A | TGCAGAAAGCAGTTTCAGATGCCAGAGTCGGAGAACCATTTTTGCAGTCTTATATGAGAGGAGGTAGAAAAACACTAGGGAGGAGAAATTGTTTTGCCA[T/A]ACCCAACAGTAGAATATGTTACTATTGAAAAAGAAATCTTCA |
| HASS_CARMENHASS_84299_GRPb_T | TGCAGAGCAGAAAAGCACATGACTTGGAACTTCAACAAGAGCCTGCATCTTGAGTCTGTC[G/T]GGAGGAACTGATCGCCTGATGGCAAATTAGGAATCTTGTTGGTGATGAGAGAGGTTTTGCCCTCCCAAATTGCATCAGTC |
| LAMBHASS_20958_G | TGCAGAATGGACCTTTTCAAACATGTAGTGGATTCTCAAATTCATGAACCCATAGAGAAGAATGAAAAGTTGGTTCTGAAGAAAGATGTAAAGAAAAAAGGA[A/G]AGCAAGGAGGTGTTTCTCACAACATGAAGCAGGTTTT |
| LAMBHASS_45259_T | TGCAGCTTCCATTTACTCTACATGCAATTTGGTTACCTCGGCTGAGAATGTATCCTGCTTTTGAGTTCTTTAGTTACAAACCCC[A/T]TGTTTATTCCAATCTTTAGAAGTGCCCTTCCACAATTATGTTTGAGAGAGAGGCA |
| LAMBHASS_93019_G | TGCAGTGAGATTGGACAGTTGATTGCTCTATGGACTATGATGATGATGCTACCC[A/G]TCCCTGTTTCAACTACCATGCACCATTACCAACCAACCCGACAACCAAAACATGATCAGACCCAGGGCCAGCAACAACGTTGTTCT |
| MALUMA_18696_C | TGCAGTAGAAGAAAGAAGGATGTAATGATCTTGGCTCCAAAGAGAAACTCTCC[T/C]TTTCTTTTCCCTCTTTTTCTCCACTTGAGAAGGAAAACCATAGTCACATCAATGAAAAATATACCTCCTTTTTTATTATTCCTGTCT |
| MALUMA_23841_G | TGCAGCTATTATTTATATCACATGATTTTTTCCATTCTATCAGGCGTTGGAGAAAACCCATCACCTGAAAGCAAGAAT[A/G]CATTACATTAGTCTACATCCAGTTTAGCCTGAGTGGGCCCCGCTATTGAGTGATCCAACTCA |
| MALUMA_82866_G | TGCAGCCTGGAGCTGTTGCTGTTATAGTTGTGTTTTGAGAGTGCGGCGAGGGAAGGACACAGCAGACAAGAGTACAGACTAGACGAAACTCAAAACCCTCGGG[A/G]CAAATGGCTGTGTGTTTTCCCCATTGCATTGCATTG |
| MALUMA_93949_T | TGCAGGATGCTGCATCATGTGTGCTGTTTTCAGTTGATAGGATAGAATAATAGT[C/T]TTTGGAGTCATGTTATTCAGAGTGACCTGCTCCATATTCTCTTGCTTTCATGGTATGGATACCAATCCAGAGTAGTTACGACCTGTAT |
| REED_31271_A | TGCAGCTCTCTCCTCTCCTCTCCTCTCTCCTCAACCAGGGCTTAGAAACCCCATTGGGTATGGGGTCAGACATTGGTCCAGGCAATAAA[T/A]AAATGGCCTCAGGCATAGGCTCATGTGAGACTGGCCTGAGTCCAGGCTCA |
| REED_81676_C | TGCAGGGCCCAGAAATGTACCAAACGCCACACGAGTACTGTTTTGTGGTGATGTTCGTGACCCCACGTTTTCGGC[T/C]CTCTGCAACTACGATCGAGCTAAATATTTGAGAAGGAAGATGCTTATTTGAGTTGGAACATGCA |
| REED_92177_T | TGCAGCATTTTCATAGTATAATTTGACATATTCCAACAATCGCTGTAG[A/T]GCCCATGGAGCAATTGGCATCAGAGGGGCTTTCAGCCCAATGAAATCCTGGCCAGGAAATTGATCTTTTTGTTGGAATCTACTATTTCGGTT |
| SHARWIL_116939_G | TGCAGTGCTCAATGCCTGCTGACACCTGTCCATGCCACGTGGCTCTCCTAAAAGTCCAAAAATTTCCACCC[T/G]TCAATCAATGCATCTTATGCAATTCATGCAATTAGATACTTGGACTATGTGGCCCATTTGAGGTTACA |
| SHARWIL_32517_A | TGCAGCTAATTGAGACAAAAGCAACCTGAAAAAAGAGAGAAAACAGAGACTAGG[T/A]CCATTTCTTTATTACAACTATACAGTAAGTTCTGATGTTGTGCTCTGAACATCATCTAGCCATGGCCCATCATGTGATTGGTTGGG |
| SHARWIL_43380_G | TGCAGTCATGGCCGGAAGCTTTTCGGGAGGTTGGCAACCTTGAAA[A/G]GACAAGTCTAGCAAGTGGAAACAATCAAGAGCCAGATGTCCAAATCGATTGCAGATCTGACACAAAGGTCTTGGCTTGGATGATGGAGATTGGG |
| SHARWIL_67950_C | TGCAGTGAAAGGCCCGAATGTTTACGGACAGTCGGAGACCCCTCTGAATG[T/C]CTGACATTTCTTGTACACCCTTGGAGAGCCTTTGACAAATGCACATACACCTTTTGACAAAAACATCATATTTTGTATGGCCTAATATTCA |
| SHEPHARD_111476_C | TGCAGGCCAAGCCGAAACTGAGCTCAAGGGAAAGCGTGGAGGAGGTGAAAAGGAAAAGCGTGCTAAAAAAAAGCAGGTCT[G/C]CAACTGTATATTCTTTATGTTCTTCATAGAGTATCTTTTCCATGGATCCTTGACTCCTCG |
| SHEPHARD_4390_G | TGCAGTTGTGCTATCTATGTGGTCCCTGCT[A/G]GCTAACTGTGTTTTATCATGTGTAGACTCTTTGGATGGTTGAGATGAGTGTGATTCTTCTACACAATTGAATGGTCAGAATTCATGAATGGTACTGGACCGGCCTAAGAT |
| SHEPHARD_91293_T | TGCAGCAAAGCATCACGGTGCCTTCATTTGCCCGTGTCTATATTTGGATGCCAAATTTTTATAGCAGTTAGAAGCACTGATAACAGCAACCAAA[C/T]AAATAATCTGGTGCATACAGATAAAATACAACCCAGGATATCTAC |
| VELVICK_94477_G | TGCAGGGCCATCAATGAGGAAACATTTGATTGAGGAAAGGCTTATCCTCTTGGAATACTTTATCAAATATA[T/G]TCTCCCCAATCTTGTAGTTGAAGAAGGTTATAATGATATCATCACTTGGGTGGGCAAAGAAGATTTCC |
| VELVICK_94905_C | TGCAGGATGTGTTGGCCATATGGAGGACTACTTGGACCAACGAATGCATGGTAGGTGGCGTGCAACT[G/C]CCTAAGACAAACTGCCCAGCAGATGGTTATTTCCAGCAAAGCCTTGGCAATTATTCAGAGGGCCAAGTTGGTA |
| VELVICK_9837_G | TGCAGCGTGTGTCCTTCCATCAAGCGTGCCATCACGCAAGACTCTAGTTGCAGGAAAAATAATGTCTTCAGGAAATGCTTGCATTGAGATCTCAGTATCCAAAAA[A/G]AGATTAGCCAAGGCCCATGTTGCTGATTCTGCAA |
| WURTZ_21778_C | TGCAGCAGCAAAAGAAGTCCCACAACAACAGCAAAAGTGAAATAGCCATAGCCCATGACTGGGAGACT[T/C]GTACCAGTCAAGGTAACTGATTTTTTTTGAGGCCATAGTCTTTCCCATTCATTTTCTTCCTCAACTCCAACA |
| WURTZ_2603_C | TGCAGTGTTTCTTCAACCAACGACTTCAAGATTTACCGAATAGACACTTCTTGGCTTGCAAATAAAGCGTTTGTTTTGGATAAGGAGAGAACAGTGAAAA[T/C]GACAACGTAGACATAGCATTTAGTTTAGTTTTCTTCGGTT |
| WURTZ_61596_A | TGCAGCACCACTTTTCTCCTCCTTCTTCTTCTTTTCTTCTTCTCTTCTTTCTTCTCTTTTCCTTCCCCTCTTCTTTTTTTCTTTCTTC[C/A]CTTTTTCTCGATCCTTCTGTTTCAGGAGAAGAAGTAGGGCAACAAAAGCCTC |

| **TABLE S2** MassARRAY assays developed for avocado cultivar identification | | | | |
| --- | --- | --- | --- | --- |
| Cultivar | Assay ID | Private Allele | Alternate Allele |  |
| Fuerte | A1 | C | T |  |
|  | A2 | C | T |  |
|  | A3 | T | C |  |
| Hass/Carmen Hass | A4 | A | T |  |
|  | A5 | T | T |  |
| Lamb Hass | A6 | G | A |  |
|  | A7 | T | A |  |
|  | A8 | G | A |  |
| Maluma Hass | A9 | C | T |  |
|  | A10 | G | A |  |
|  | A11 | G | A |  |
|  | A12 | T | C |  |
| Reed | A13 | A | T |  |
|  | A14 | C | T |  |
|  | A15 | T | A |  |
| Sharwil | A16 | G | T |  |
|  | A17 | A | T |  |
|  | A18 | G | A |  |
|  | A19 | C | T |  |
| Shepard | A20 | C | G |  |
|  | A21 | G | A |  |
|  | A22 | T | C |  |
| Velvick | A23 | G | T |  |
|  | A24 | C | G |  |
|  | A25 | G | A |  |
| Wurtz | A26 | C | T |  |
|  | A27 | C | T |  |
|  | A28 | A | C |  |
| Assay primers and sequence information available in Supplementary Material S1. | | | | |

Avocado Microsatellite Marker Analysis

DNA of each sample was ampliﬁed at 15 microsatellite loci that were suitable for building a reference database of the eight potential pollen parents present in a 5-km radius around each orchard (Sharon et al. 1997; Borrone et al. 2007).

PCR conditions

The 5′ end of each forward primer was fluorescently labelled (Table S3). PCR was performed using the Qiagen Type-it Microsatellite PCR Kit. Three multiplex reactions were performed for each sample, in 12.5 μL reaction volumes containing approximately 5 ng DNA template, 2x Type-it Multiplex PCR Master Mix, 0.2 μM of each oligonucleotide primer, 1.6 μg BSA and 0.75 μL sterile water. PCR was performed with initial denaturation at 95°C for 5 min, followed by 32 cycles of 95°C for 30 s, 57°C for 90 s and 72°C for 30 s, followed by final elongation at 60°C for 30 min. Marker AVAC01 failed in the multiplex PCR. PCR for this marker was performed in 12.5 μL reaction volumes containing approximately 5 ng DNA template, 1 × F1 Taq reaction buffer, 0.02 U F1 Taq (Fisher Biotec), 0.1 mM dNTPs, 2.2 mM MgCl_2_, 0.002 mg BSA, 0.2 μM of each oligonucleotide primer and 6.7 μL sterile water. PCR was performed with initial denaturation at 94°C for 2 min, followed by 32 cycles of 94°C for 20 s, 40°C for 45 s and 72°C for 1 min, followed by final elongation at 72°C for 10 min. Genotypes were generated using an AB 3500 Genetic Analyser (Applied Biosystems, Foster City, CA) and allele sizes scored relative to an internal standard (600 LIZ® Size Standard, Applied Biosystems, Foster City, CA) using the program GeneMarker version 2.6.3 (SoftGenetics, State College, PA).

Cervus settings

The pollen parent of each fruit was assigned using the software CERVUS version 3.0.7 (Kalinowski, Taper & Marshall 2007).Simulations on paternity were run using 100,000 iterations assuming 0.99 of candidate fathers were sampled and 0.01 proportion of loci mistyped. CERVUS calculated a likelihood ratio that represented how much more likely a putative pollen parent was compared with a pollen parent selected randomly from the reference library. The likelihood ratio was expressed as the LOD score, which is the natural logarithm of the likelihood ratio. We only considered pollen parents possible if CERVUS assigned a positive LOD score. Paternity was assigned to the most likely pollen parent with statistical confidence at strict, 95%, and relaxed, 80% levels. We assigned a specific pollen parent if Cervus assigned a positive LOD score to one candidate father only, or if Cervus assigned a positive LOD score and the best match met the 95% strict-confidence level (based on 100,000 iterations).

| Table S3 Characterisation of 15 polymorphic microsatellite loci used to determine paternity of avocado seeds (Sharon et al. 1997; Borrone et al. 2007) | | | | |
| --- | --- | --- | --- | --- |
| Locus | **Primer sequences (5' to 3')** | **Repeat motif** | **Fluorescent label** | **Allele sizes** |
| AVAC01 | F: CTGGTTGCTCTCTTGTCTACATAATA | (AG)_15_ | VIC | 97–154 |
|  | R: CGGTTTTGTAAGTTGATAG |  |  |  |
| AVAG03 | F: GCACTTCCTAAACTTGCAGGT | (TC)_17_ | FAM | 95–103 |
|  | R: CTGAACATCCAATGACAAACATCC |  |  |  |
| AVMIX03 | F: GATATTCCTGTTGTCACTGC | (TG)_16,_ (AG)_20_ | PET | 145–173 |
|  | R: AATGTTCCCCATGAAAGTCTCC |  |  |  |
| AVAG05 | F: GGATCTGATGTGTGGGGGAG | (AG)_10_ | PET | 92–98 |
|  | R: CCTGTCGGAAAAGACTATGCG |  |  |  |
| AVAG06 | F: CGACCTCTTCTTATACTC | (CT)_18_ | NED | 70–83 |
|  | R: GTACCTCTGATAATGAGCAT |  |  |  |
| SHRSPa010 | F: CGAAGAAGGATAGTCTGAAAACCC | (AAC)_7_ | VIC | 131–134 |
|  | R: GAGGAAGGATCGGAAGAGAGG |  |  |  |
| AVAG22 | F: GATCATCAAGTCCTCCTTGG | (GA)_15_ | VIC | 101–125 |
|  | R: GATCTCATAGTCCAAATAATGC |  |  |  |
| AVAG25 | F: ATGGTTTTTTCCTGCCCTTT | (TC)_14_ | NED | 101–111 |
|  | R: AACAAGCCCCCTAAAAGAA |  |  |  |
| SHRSPa033 | F: TGCCAAGTGAGACTCAATTCC | (CT)_14_ | FAM | 93–103 |
|  | R: CAAATCCTTCCACTGACCAAC |  |  |  |
| SHRSPa056 | F: GGTTTTCCAATTCTCTCTATCCCC | (CT)_14_ | FAM | 91–115 |
|  | R: TTGATGCCTGCTTCCGTGTG |  |  |  |
| SHRSPa057 | F: GCAAGGCATTACGATGTCA | (CT)_16_ | NED | 188–208 |
|  | R: CTCTAGTGGACAAAATCGACAA |  |  |  |
| SHRSPa081 | F: GGGCTTCAATTCAATCCAATCC | (C)_2_(GA)_7_ | PET | 214–250 |
|  | R: TCTTCAGCACGCCACGAGTCT |  |  |  |
| SHRSPa089 | F: GCAGAAGCCACCTCAGAAC | (CT)_14_A(TG)_3_(CG)_2_ | VIC | 294–328 |
|  | R: CAAACAACAGATGAGCGGA |  |  |  |
| SHRSPa093 | F: CGACGAAAACAGCAAGAGGAG | (TC)_22_ | PET | 305–328 |
|  | R: TCAATCAACCACAAAGGGACTATG |  |  |  |
| SHRSPa111 | F: ACCTTTCAATCTCTTCCCAAAC | (AG)_8_G(GA)_5_ | VIC | 151–172 |
|  | R: CCGCTCCAATCCAACCA |  |  |  |

Kalinowski, S.T., Taper, M.L. & Marshall, T.C. (2007) Revising how the computer program CERVUS accommodates genotyping error increases success in paternity assignment. *Molecular Ecology,* **16,** 1099-1106.
